# Web-Based Cognitive-Behavioral Therapy to Reduce Severe Cancer-Related Fatigue Among Survivors of Hodgkin Lymphoma: A Feasibility Study

**DOI:** 10.1007/s10880-023-09944-6

**Published:** 2023-02-20

**Authors:** Peter Esser, Horst Müller, Peter Borchmann, Stefanie Kreissl, Hans Knoop, Uwe Platzbecker, Vladan Vucinic, Anja Mehnert-Theuerkauf

**Affiliations:** 1 Department of Medical Psychology and Medical Sociology, University of Leipzig, Philipp-Rosenthal-Straße 55, 04103 Leipzig, Germany; 2 German Hodgkin Study Group (GHSG), Clinic I for Internal Medicine at the University Medical Center of Cologne, Cologne, Germany; 3 Department of Medical Psychology, Amsterdam University Medical Centers, University of Amsterdam, Amsterdam Public Health Research Institute, Amsterdam, Netherlands; 4 Clinic for Hematology, Cellular Therapy and Hemostaseology, University Medical Center Leipzig, Leipzig, Germany

**Keywords:** Hodgkin lymphoma, Cancer-related fatigue, Cognitive-behavioral therapy, Online interventions, Survivorship

## Abstract

We investigated the feasibility of a web-based cognitive-behavioral therapy to reduce cancer-related fatigue (CRF) among survivors of Hodgkin lymphoma. In this before-and-after trial, patients were primarily recruited via the German Hodgkin Study Group (GHSG). We assessed feasibility (response and drop-out rate) and preliminary efficacy including CRF, quality of life (QoL), and depressive symptomatology. *T* tests compared baseline levels with t1 (post treatment) and t2 (3 months of follow-up). Among 79 patients contacted via the GHSG, 33 provided interest (42%). Among the seventeen participants, four were treated face-to-face (pilot patients), 13 underwent the web-based version. Ten patients completed the treatment (41%). Among all participants, CRF, depressive symptomatology, and QoL improved at t1 (*p* ≤ .03). The effect in one of the CRF measures remained at t2 (*p* = .03). Except for QoL, post-treatment effects were replicated among the completers of the web-based version (*p* ≤ .04). The potential for this program has been demonstrated, but needs to be re-assessed after identified issues on feasibility have been resolved.

Trial registration: The study was registered at ClinicalTrials.gov (Number: NCT03968250).

**Supplementary Information:**

The online version contains supplementary material available at 10.1007/s10880-023-09944-6.

## Introduction

Cancer-related fatigue (CRF) is defined as a persistent feeling of strong exhaustion that cannot be resolved by rest (National Comprehensive Cancer Network® 2015) and is one of the most frequent and distressing symptoms among cancer survivors (Bower et al., 2014). Among survivors with Hodgkin lymphoma (HL), a systematic review including 22 studies found prevalence rates of CRF in up to 74% of patients (Daniëls et al., 2013), and a large study (*n* = 4215) showed that CRF persists even 5 years after treatment among this patient group (Kreissl et al., 2016).

CRF among HL survivors is associated with negative effects on both employment and financial issues (Behringer et al., 2016), which may be particularly relevant among this population with a median age of around 30 years at diagnosis (Hochberg et al., 2009; Shanbhag & Ambinder, 2018). A longitudinal study on quality of life (QoL) in HL survivors (*n* = 4215) revealed clinically relevant long-term impairments in various aspects of QoL such as physical, role, and social functioning (Kreissl et al., 2020).

Since the etiology of CRF is assumed to be multi-dimensional (National Comprehensive Cancer Network® 2015), different therapeutic approaches have been evaluated. Pharmacologic treatment with stimulants may help in advanced stages and during oncological treatment, but is not recommended for persistent CRF among survivors in remission (Bower et al., 2014). Furthermore, a meta-analyses based on 113 articles compared pharmacological and non-pharmacological interventions aimed at CRF and concluded that psychological and exercise treatments are superior to pharmacological interventions (Mustian et al., 2017).

Based on theoretical and empirical knowledge, a Dutch research group developed a cognitive-behavioral therapy (CBT) program to treat CRF. The treatment concept assumes that CRF is caused by cancer and its treatment, but is maintained by perpetuating factors which can be addressed by CBT. These factors include insufficient processing of the fact that one had cancer and was treated, dysbalanced activity level, dysregulated sleep–wake rhythm, CRF-related dysfunctional cognitions, fear of cancer recurrence, and negative social interactions (Gielissen et al., 2006). The perpetuating factors are represented in specific treatment modules which are individually selected for each patient based on their needs. The face-to-face version consists of approximately twelve sessions of about 50 min across six months (Gielissen et al., 2006). In the online version, face-to-face contacts are limited to the start and end of the treatment (Abrahams et al., 2015, 2017), whereas communication during the intervention runs via a specific online program. Randomized clinical trials (RCT) have demonstrated the efficacy of both the face-to-face (Gielissen et al., 2006) and the web-based version (Abrahams et al., 2017; Janse et al., 2018; Jim et al., 2020).

Despite these promising results, the program is not yet available in German. Furthermore, it is unclear if the program would benefit HL survivors with CRF as previous evaluations are based on survivors with breast cancer (Abrahams et al., 2017; Gielissen et al., 2006), palliative cancer patients (Poort et al., 2020), and patients with chronic myeloid leukemia (CML) (Jim et al., 2020). Given the unique characteristics of HL survivors compared to other cancer populations, e.g., relatively young age and good curability (Hochberg et al., 2009; Shanbhag & Ambinder, 2018), they are in need of interventions to improve their long-term quality of life.

To address the research gaps outlined above, we translated and adapted the program and subsequently conducted a pilot trial to (i) assess feasibility of both the CBT program and the trial design and (ii) investigate preliminary efficacy in reducing levels of CRF and improving QoL. With this study, we aimed at preparing the ground for a future trial to assess robust data on efficacy.

## Methods

### Design

We conducted an interventional before-and-after phase II trial. Primary outcomes included measures of feasibility, secondary outcomes included measures to analyze preliminary efficacy. The study was conducted by (i) the Department for Medical Psychology and Medical Sociology at the University Medical Center of Leipzig and (ii) the German Hodgkin Study Group (GHSG) placed within the Clinic I for Internal Medicine at the University Hospital of Cologne. It was supervised by a member of the research group which developed the treatment (HK) based in the Department of Medical Psychology at the Amsterdam University Medical Centers.

All participants provided written informed consent before study participation. The study was approved by the Ethics Committee of the Medical Faculty of the Universities of Leipzig (file number: 501/17-ek) and Cologne (file number: 18-247) and registered at ClinicalTrials.gov (number: NCT03968250).

### Recruitment and Screening Procedure

The majority of patients were recruited via the GHSG network. The network is centered in Cologne and cooperates with hematological clinics and practices across Germany to conduct nationwide clinical trials with the aim of improving diagnostic, treatment, and follow-up care of HL.

One study of the network is dedicated to long-term QoL of HL survivors. Using the EORTC QLQ-C30 (Aaronson et al., 1993), these patients are regularly assessed from diagnosis up to 5 years post treatment for CRF and other domains of QoL. The available data on CRF enabled us to select patients with a fatigue symptom scale score > 30 as the intervention was designed for survivors with high levels of CRF. A second requirement for selection was that patients lived in an area which was close or had good traffic connections to one of the two study centers since two treatment sessions and the screening procedure was conducted in person. Patients meeting both requirements were contacted by an invitation letter; those who provided interest via a letter in response were called by phone to be provided with more study details.

Consenting patients first underwent an online ‘pre-screening’ to preclude patients with violations in CRF or depressive symptomatology (see eligibility criteria) before they received the in person screening. For assessing depression, patients exceeding the cut-off (> 10) on the PHQ-9 (Martin et al., 2006) were further diagnosed by the depression module of the SCID-IV (First & Gibbon, 2004). The subsequent in person screening was conducted at the respective study center and consisted of a psychological assessment by the respective study coordinator and a medical screening by a hematologist.

Due to low enrollment within the first phase of recruitment, we widened recruitment sources during the study to enlarge the pool of potential participants. In detail, interested patients were referred by physicians from hemato-oncological clinics and private practices, psycho-oncologists at a psychosocial counseling center, and physicians who were informed about the study at a scientific congress on hematological diseases.

### Eligibility Criteria

As another measure to enlarge the pool of potential participants, we decided to widen eligibility criteria by including patients (i) with relapse and (ii) with depression and/or psychotropic treatment if certain conditions were met. The final criteria were as follows:

Patients were included if they were (i) diagnosed with Hodgkin lymphoma (ICD-10: C81), (ii) in complete remission for at least 12 months (first diagnosis or relapse), (iii) ≥ 18 years old, (iv) fluent in German language, (v) able to access the internet and use the online program, (vi) meeting the criteria for CRF, i.e., if the clinical interview with a psychologist confirmed that the fatigue symptoms result from HL and/or its treatment and that these symptoms severely impair various areas of functioning daily life such as work, family, physical tasks, or social life, and (vii) having a sum score ≥ 35 on the CIS-Fat within the screening process. Patients were excluded in case of (i) major communication difficulties, (ii) severe cognitive impairment interfering with ability to give informed consent, (iii) a Karnofsky Index < 70 predicting an expected survival of less than 6 months, (iv) somatic co-morbidities that could explain the presence of CRF such as hypothyroidism or chronic obstructive pulmonary disease, (v) clinical symptoms which may indicate a relapse of HL, (vi) current treatment aimed at CRF such as activity programs or medical substances, (vii) previous attempt to treat CRF with another CBT program, (viii) current depression or antidepressant medication if the depression was considered the major cause of the fatigue symptoms or if the depression was supposed to hamper treatment completion, or (ix) current medical or psychological treatment for a psychiatric disorder other than antidepressant medication.

### Translation and Adaptation

We translated (i) the treatment manual outlining the general concept and the treatment procedure for the face-to-face version and (ii) the online content for the web-based version which is presented to the patients within the online program. All content was translated from Dutch into German by a German psychologist who had graduated in the Netherlands; the translations were subsequently re-checked by one of the study therapists who also had graduated in the Netherlands in terms of wording (e.g., correct translation of psychotherapeutic technical terms) and correctness of instructions and descriptions. We also translated the questionnaires assessing the perpetuating factors if no German translation or equivalent instrument existed (see appendix, Table S1).

Adaptation to HL survivors was largely guided by a well-established approach (ADAPT-ITT) (Wingood & DiClemente, 2008). We conducted semi-structured interviews with two experts (one hemato-oncologist and one psycho-oncologist) and two HL survivors suffering from CRF. Having discussed the results with HK, the structure of the program was considered adequate for this population and thus could be maintained. For the online presentation of the web-based version, we used the Dutch template in which we filled in the translated content. Within the online program, we selected images whose persons were relatively young to reflect the average age of HL survivors; videos on experience reports of Dutch participants were subtitled in German.

### Intervention

The web-based treatment consisted of a maximum of eight web-based modules to set the treatment goals (module 1), to address the perpetuating factors, i.e., insufficient coping, dysbalanced activity level, dysregulated sleep–wake rhythm, CRF-related dysfunctional cognitions, fear of cancer recurrence and negative social interactions (modules 2–7), and to realize the treatment goals (module 8). In addition to the online work, two face-to-face contacts with the therapist were needed at the start and the end of the treatment, respectively. The intended treatment time was six months.

At intervention start, therapists and participants met face-to-face to establish a therapeutic relationship and to select the treatment modules according to their individual needs using a set of questionnaires (see Table S1) and a clinical interview. Subsequently, the treatment goals were set (module 1) before the work on the individualized selection of the modules 2–7 started. After the work on the perpetuating factors was completed, the treatment goals were finalized (module 8). At the end of the treatment, the second face-to-face session took place to evaluate and complete the treatment.

For providing information on the web-based modules and for communication between therapists and patients, we used the Dutch treatment template based on the content management system *MindDistrict*. During the study, it turned out that some patients had problems to handle the program or found it hard to follow instructions via mail without a personal contact. Therefore, telephone contacts were introduced during the study. The use of such phone calls were discussed with the supervisor on a case-by-case basis.

For training reasons (see section below), the first four recruited patients were treated completely face-to-face across approximately twelve treatment sessions. The screening procedure, the content, and the structure of the treatment was equal to the web-based version.

### Training and Supervision

The initial therapists (two in Leipzig and two in Cologne) were trained within a three-day workshop by HK including role plays with actors. For training reasons, the first four recruited patients were treated face-to-face in order to collect practical experience with the content of the treatment manual before learning the additional therapeutic techniques to conduct the online treatment. Since the start of the first treatment, two-weekly telephone supervisions guided by HK were held during the whole treatment phase. Before the therapists started to work with the online treatment, another half-day online workshop was held by HK to introduce the therapists into the specifics of the online program such as writing of therapeutic E-Mails and handling the online program. During the study, both therapists in Leipzig were not available for a longer time period. Therefore, a new therapist was introduced into the treatment program and started to join the supervisions before she took over the two remaining patients.

### Outcomes

#### Primary Outcomes (Feasibility)

We assessed response and drop-out rate as well as patient satisfaction with the Working Alliance Inventory—Short Revised (WAI-SR) (Munder et al., 2010) and the German patient satisfaction questionnaire (ZUF-8) (Schmidt et al., 1989). The two questionnaires were slightly adapted: Items in present tense within the WAI-SR were changed to past tense and the term ‘clinic’ within the ZUF-8 was replaced by ‘institution.’

#### Secondary Outcomes (Preliminary Efficacy)

We assessed CRF with two instruments. The CIS-Fat (Worm-Smeitink et al., 2017) is the Fatigue subscale of the Checklist Individual Strength (CIS) and consists of 8 items with a 7-point Likert Scale (possible range: 8 to 56). The EORTC Quality-of-Life Questionnaire Module on Cancer-Related Fatigue (FA-12) consists of 12 items rated on a 4-point Likert Scale and assesses the physical (5 items), cognitive (3 items), and emotional (2 items) components of CRF. Additionally, a sum score across all twelve items can be generated (Hinz et al., 2018). All scores are transformed to values ranging from 0 to 100. Higher scores in the CIS-Fat and the FA-12 indicate higher CRF. QoL was assessed with the EORTC QLQ-C30 global quality-of-life subscale (Schwarz & Hinz, 2001) based on two items on a 7-point Likert scale. Values are transformed on a scale ranging from 0 to 100, with higher values indicating higher QoL. The Patient Health Questionnaire-9 (PHQ-9) (Martin et al., 2006) assesses depressive symptomatology on 9 items with a 3-point Likert scale (possible range: 0–27). Higher values indicate higher levels of depressive symptomatology.

#### Administration of Outcomes

Outcomes assessing preliminary efficacy were assessed pre-intervention (t0), post-intervention (t1), and 3 months after t1 (t2). The outcomes on treatment satisfaction were assessed at t1. Questionnaires at t0 and t1 were completed in the respective treatment institutions, t2-questionnaires were sent by mail to be completed at home.

### Statistical Analyses

Descriptive analyses were used to provide sample characteristics.

Response rate was defined as the number of patients providing initial interest on the letter in response divided by the number of contacted patients with a valid address. Therefore, response rate could only be calculated for the patients which were contacted by mail via the GHSG network. Drop-out rate was calculated as the number of study completers divided by the number of patients that started the treatment. For both measures on treatment satisfaction, we calculated means and subsequently applied t-tests for independent samples to contrast these means with reference values in order to check to which degree these values are comparable to patients treated with other psychological treatments. For the ZUF-8, we used a reference value of psychosomatic patients in a face-to-face inpatient rehabilitation (*n* = 15,702) (Kriz et al., 2008); for the WAI-SR, we used data from 88 outpatients treated with face-to-face psychotherapy (Munder et al., 2010).

To assess preliminary efficacy, *t *tests for dependent samples were used to compare the levels in the outcomes at t0 with those at t1 and t2, respectively. Due to small sample size, we first ran intent-to-treat analyses based on all study participants (i.e., also including drop-outs and those with face-to-face treatment) to obtain maximum statistical power.

To identify the potential of the online program, we additionally applied per-protocol analyses including only those patients who both received and completed the online program. Since almost all t2-assessments fell within the COVID-19 lockdowns in Germany, we only compared t0 and t1 to avoid bias of the treatment effects.

The alpha level was set at 0.05. Cohen´s *d* was provided as effect size, with *d* of 0.20, 0.50, and 0.80 being interpreted as small, medium, and large effects, respectively (Cohen, 1992). Sum scores of the outcomes were generated if more than 50% of the respective items were completed. We conducted analyses with SPSS, Version 27 (IBM Statistics).

### Deviations from Initial Study Protocol

A major deviation refers to the widening of the recruitment sources and eligibility criteria to enlarge the patient pool (see above). We first amended the recruitment procedure and subsequently adapted eligibility criteria since the recruitment issues remained. Another major deviation was the introduction of telephone contacts if considered necessary for effective treatment (see above). For minor deviations not outlined in the manuscript see Table S2.

## Results

### Enrollment and Drop-Out Rate

**Fig. 1:**
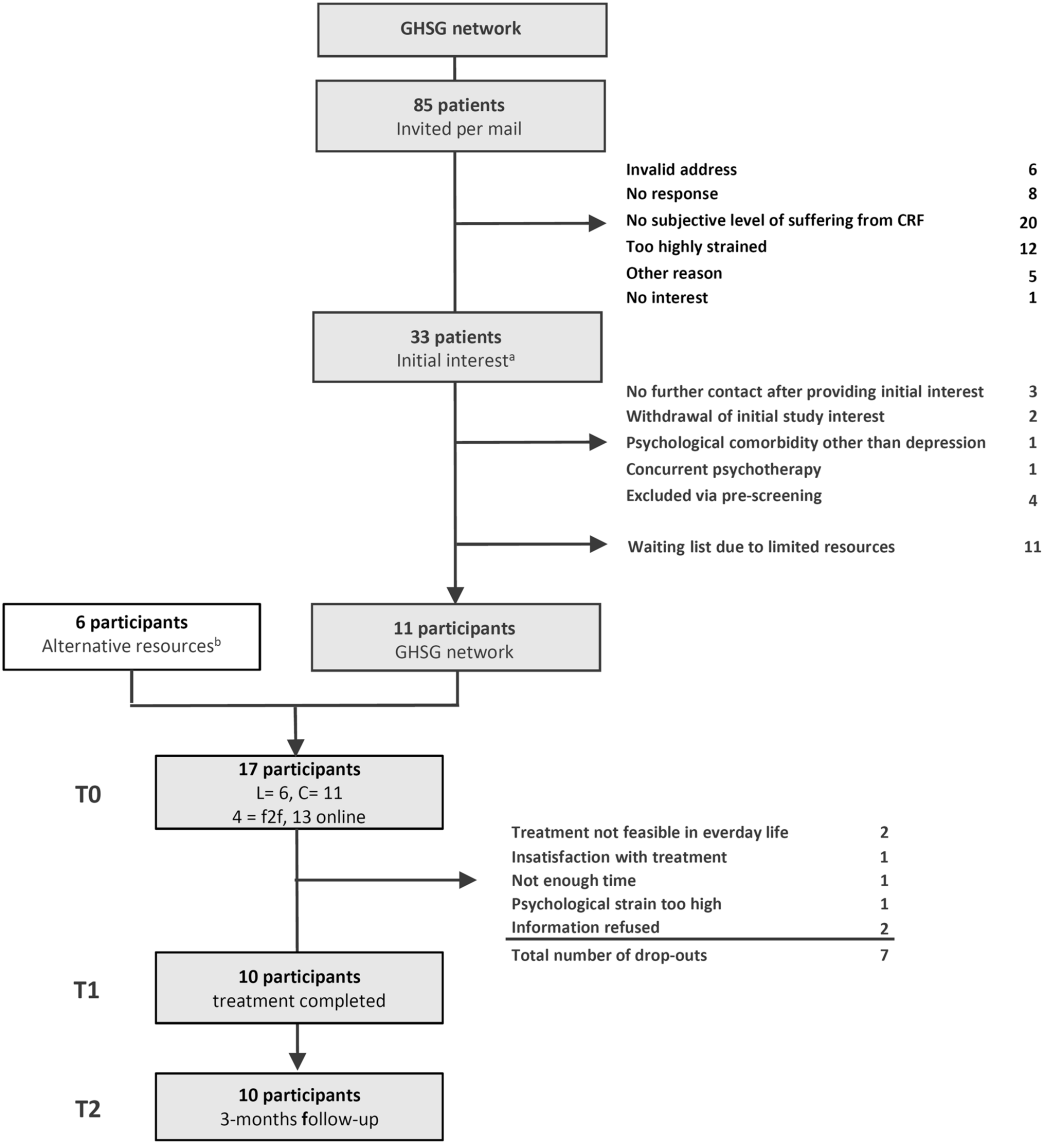
Flowchart. GHSG, German Hodgkin Study Group; L, study center Leipzig; C, study center Cologne; f2f, face-to-face treatments among the pilot patients; ^a^all patients who provided interest via the pre-stamped letter in response. ^b^several sources were approached including hematological clinics, congresses, and self-help groups

Between September 2018 and March 2020, 85 patients from the GHSG network were contacted (Fig. 1). Among those with valid address (*n* = 79), 33 patients provided initial study interest (response rate: 42%). Among these 33 patients, 11 participants were finally included. Six further patients could be recruited via other sources, resulting in a final sample of 17 participants.

Of the 17 patients who started the intervention, 10 patients completed the program (drop-out rate: 41%). Among those who discontinued, three patients cited the program itself as their reason (i.e., content was difficult to apply in daily life; patients were unsatisfied with the content). Two patients discontinued for personal reasons (lack of time; excessive mental burden).

### Formal Treatment Information

Four patients were treated face-to-face, 13 patients underwent the online version. Among the 10 study completers, mean treatment duration was 8 months (range: 5–14 months).

### Baseline Sample Characteristics

Nine patients (53%) were female. The mean age was 47 years, ranging from 29 to 63 years (Table 1). The mean time since diagnosis was 6 years, and almost one-third had been treated with stem cell transplantation. More than half (*n* = 11) reported clinical levels of depressive symptomatology.

**Table 1.** Sample characteristics (*N* = 17)

	*N* (%)
Gender	
Male	8 (47)
Female	9 (53)
Age (M, SD)	47 (11)
Employment	
Fully working	7 (41)
Partially working	4 (24)
Disability pension	2 (12)
Regular pension	2 (12)
Other	2 (12)
Years since initial diagnosis (M, SD)	6 (3)
History of relapse	8 (47)
Treatment of HL^a^	
Polychemotherapy^b^	17 (100)
Radiotherapy	3 (18)
Surgery	1 (6)
Autologous stem cell transplantation	4 (24)
Allogeneic stem cell transplantation	1 (6)
Clinical level of depression^c^	11 (55)

^a^Multiple answers possible

^b^Conventional and PET-adapted polychemotherapy with ABVD and/or BEACOPP in the GHSG trials HD16-18

^c^PHQ-9 ≥ 10 according to guidelines of the American Society of Clinical Oncology

### Patient Satisfaction and Treatment Adherence

The mean treatment satisfaction was 21 (SD: 1.5) and thus significantly below the reference value of 25 for face-to-face psychosomatic treatments (*p* < 0.001). The mean evaluation of the working alliance between patients and therapists was 4 (SD: 0.8) and thus close to the reference value of 3.8 found in psychotherapy outpatients (*p* = 0.29).

Among the 9 patients who commented on a free text field, 6 patients reported difficulties in handling the online tool; nevertheless, these problems were not reported to be a reason for drop-out.

Adherence to the treatment protocol was closely monitored by HK within the supervisions.

### Preliminary Efficacy

Intent-to-treat analyses revealed significant improvements post treatment (t0 vs. t1): Total scores in both CRF instruments and depressive symptomatology decreased, while levels of QoL increased (Table 2). Across the subscales of the FA-12, only the physical component significantly decreased. Effects for the significant improvements ranged from medium to large. At three months of follow-up (t0 vs t2), significant improvements remained in the CRF total score of the CIS-Fat and in the physical component of the FA-12 (Table 2), with medium to large effects.

**Table 2.** Intent-to-treat-analyses^a^

Baseline vs. post treatment	*N*	*M (SD)*		*d*	*p*
		T0	T1		
Fatigue (FA-12)					
Total score	15	46 (13)	35 (15)	.71	.02
Physical component	15	68 (21)	48 (23)	.81	< .01
Emotional component	15	27 (21)	27 (23)	.00	1.0
Cognitive component	15	23 (14)	17 (18)	.31	.25
Fatigue (CIS-Fat)	15	41 (8)	32 (12)	.63	.03
Quality of life	15	50 (17)	63 (16)	.65	.02
Depressive symptomatology	15	9 (3)	6 (3)	.77	.01

Baseline vs. 3 months of follow-up	*N*	*M (SD)*		*d*	*p*
		T0	T2		
Fatigue (FA-12)					
Total score	10	50 (11)	44 (21)	.37	.27
Physical component	10	75 (19)	62 (27)	.66	.04
Emotional component	10	27 (22)	31 (28)	−.12	.89
Cognitive component	10	23 (12)	25 (14)	−.11	.29
Fatigue (CIS-Fat)	10	44 (6)	37 (14)	.85	.03
Quality of life	10	54 (14)	50 (23)	.14	.69
Depressive symptomatology	10	10.1 (2)	9.7 (4)	.11	.07

N, included cases; M, mean; SD, standard deviation; d, Cohen’s d

^a^Including all patients irrespective of type of delivery (online vs. face-to-face) and study completion

Per-protocol analyses revealed a significant decrease in both total CRF scores, the physical CRF component of the FA-12 and depressive symptomatology (Table 3). There was no significant improvement in QoL. Effects for the significant improvements were large.

**Table 3.** Per-protocol analyses^a^

Baseline vs. 3 months of follow-up	N	M (SD)		d	*P* (*t *test)
		T0	T1		
Fatigue (FA-12)					
Total score	8	48 (11)	32 (18)	1.31	< .01
Physical component	8	74 (20)	43 (24)	1.85	.001
Emotional component	8	24 (23)	25 (26)	−.06	.88
Cognitive component	8	21 (12)	21 (23)	.00	1.0
Fatigue (CIS-Fat)	8	44 (7)	28 (9)	4.62	< .001
Quality of life	8	51 (20)	64 (15)	−.48	.22
Depressive symptomatology	8	10 (3)	6 (4)	.89	.04

*NI* included cases; *M* mean; *SD* standard deviation; *d* Cohen´s d

^a^Including all completers of the online version

## Discussion

### Main Findings

This study translated, adapted, and preliminary evaluated a cognitive-behavioral online program to reduce CRF among survivors of HL. Preliminarily results suggest that the program is effective among this population. Feasibility in terms of recruitment and attrition was notably limited.

### Interpretation Regarding Feasibility

Using our recruitment via the GHSG network, less than half of the pre-selected patients who were invited for the study participated. Comparison with previous studies is difficult since recruitment and pre-selection differed from our approach. One previous study exclusively recruited via (self) referrals (Abrahams et al., 2017), whereas another study pre-selected patients at a clinic based on the information in the medical chart and a consultation with the treating physician (Jim et al., 2020). Nevertheless, the latter study presented that only 8% of patients screened for eligibility met eligibility criteria (Jim et al., 2020) and thus supports our experience that a large patient pool is needed to reach a reasonable number of patients to strictly apply all eligibility criteria.

Previous studies found that 92% (Abrahams et al., 2017), 79% (Jim et al., 2020), and 76% (Gielissen et al., 2006) of the patients allocated to the intervention completed the minimum number of sessions. Compared to these findings, our rate of study completers (59%) was relatively low. This may be explained by the widened eligibility criteria as a result from the low enrollment: Even though we did not find an association between clinical level of depression (PHQ-9 sum score ≥ 10) and study drop-out (*data not shown*), the final sample included relatively many patients with relapsed lymphoma and invasive therapy lines such as stem cell transplantation. Accordingly, the median age in this cohort was higher than in newly diagnosed HL patients, and the study population had a longer disease history and potentially a longer time of CRF than it was originally intended for the study sample. A higher level of chronicity of CRF combined with comorbidity due to more invasive treatment may have led to higher drop-out rates as result of lack of hope and motivation, difficulties in changing chronic maladaptive behavior or physical issues limiting continuous, and effective work on the treatment modules. Therefore, it can be suggested that strict application of the initial eligibility criteria is highly needed to make treatment and future trials feasible.

The treatment satisfaction was lower than the reference value (Kriz et al., 2008), whereas working alliance was equal to the reference value (Munder et al., 2010). Since previous studies on the program did not directly assess treatment satisfaction or working alliance (Abrahams et al., 2017; Jim et al., 2020), we used reference values among non-cancer populations receiving face-to-face psychosomatic treatments and thus comparisons must be interpreted with caution. Nevertheless, the relatively low treatment satisfaction is supported by the free text comments in which participants reported issues with the online program. At the same time, results on working alliance suggest that it was possible to create an effective relationship between therapists and patients.

### Interpretation Regarding Preliminary Efficacy

Both intent-to-treat and per-protocol analyses revealed significant improvements in CRF, QoL, and depressive symptomatology between baseline and post-treatment. Looking at the different subscales of CRF, only the physical component improved. As an explanation, baseline physical CRF was considerably higher than cognitive and emotional CRF and therefore was the main complaint of our cohort.

Most treatment effects were not maintained until follow-up, which is in contrast to the previous findings on positive long-term effects of this program, e.g., at 6 months of follow-up (Gielissen et al., 2006) or demonstrated stability of treatment effects after a mean of two years (Gielissen et al., 2007). A plausible explanation for the lack of a long-term effect is that the follow-up assessments fell into the second COVID-19 lockdown in Germany. Accordingly, patients reported at follow-up to severely suffer from the lockdown situation and that working on modules such as social support could not be maintained due to restrictions.

In contrast to the initial plan, therapists introduced telephone calls during the web-based treatment if considered necessary to achieve an effective therapeutic communication. Nevertheless, synchronous communication was also a part of previous evaluation. The original evaluation of the online program allowed video or telephone sessions (Abrahams et al., 2017) and a recent adaptation study delivered the treatment completely via regular video conferences (Jim et al., 2020).

### Implications

The preliminary findings demonstrated potential for utility of this program among HL survivors and thus warrant further research. Nevertheless, the potential needs to be re-assessed after the identified issues in feasibility have been addressed within a future trial.

A main issue to make the program feasible is to modify the recruitment approach: In the current study, the two personal therapist–patient contacts and the screening at the respective study center limited the number of the patient pool to those living near to the study centers. A reasonable option to overcome this geographical limitation would be to (i) cooperate with hematologists across Germany to offer medical screenings at more locations and (ii) to conduct the face-to-face sessions via video. This strategy may enable use of the nationwide GHSG network in all parts of Germany and thus may result in a larger patient pool.

The higher number of potential patients in turn may also enable a strict application of the eligibility criteria: Focusing on those with first-line therapy and without psychological morbidity may ensure the ability and motivation to complete the treatment and thus may contribute to decreased attrition rates.

The need of telephone contacts implemented during the study may have various implications. One is that therapists need more training to effectively apply the online version. Furthermore, the inclusion of patients with psychological comorbidity may have complicated the communication via the online format and thus a strict application of eligibility criteria may resolve this issue. Nevertheless, it is to note that previous studies on the web-based version also applied synchronous communication, either by video or telephone (Abrahams et al., 2017; Jim et al., 2020). To optimize treatment acceptance, the need for regular synchronous communication within the web-based version or the possibility of a complete face-to-face treatment might be openly discussed with each patient based on individual preferences.

Finally, many patients considered it hard to use the online program. As a solution, an extra session explicitly dedicated to the technical aspects of the program may help to ensure skills and motivation from the very beginning. Rules of conduct may be established together with the patient to ensure a sustainable therapeutic alliance, e.g., fixed reaction times to comments or possibility to report the need for a telephone call.

### Strengths and Limitations

Our study provided the German translation for one of the most promising psychotherapeutic programs to reduce CRF. We also showed that the program has the potential to be effective among HL survivors, a cancer population which is rarely studied to date.

The small size and the sociodemographic and medical peculiarities of this sample, the lack of a control group and adaptations of the recruitment procedure do not allow to draw firm conclusions about efficacy. Furthermore, we did not apply forward–backward translation for the manual and the online content. Nevertheless, all translations were re-checked by a psychotherapist fluent in German and Dutch. Finally, response rate could only be calculated for patients recruited via the GHSG network and thus the findings must not be generalized for the whole sample.

### Conclusions

This study translated and applied a web-based CBT to reduce CRF among HL survivors. Preliminary data demonstrated its potential among HL survivors, which needs to be re-assessed after identified issues regarding feasibility have been addressed within a subsequent trial.

### Supplementary Information

Below is the link to the electronic supplementary material.

Supplementary file1 (DOCX 22 kb)

## Data Availability

The data and codes to analyze the data can be obtained from the authors upon request.

## Code Availability

The codes to analyze the data can be obtained from the authors upon request.
