# Supplementary material for: Web-Based Cognitive-Behavioral Therapy to Reduce Severe Cancer-Related Fatigue Among Survivors of Hodgkin Lymphoma: A Feasibility Study
Source: J Clin Psychol Med Settings. 2023 Feb 20;30(4):856–65. doi: 10.1007/s10880-023-09944-6 (PMC9943038; doi:10.1007/s10880-023-09944-6)
Supplement: Supplementary file 1 — Supplementary file1 (DOCX 22 kb) [file 10880_2023_9944_MOESM1_ESM.docx]

| **Table S1.** *Instruments and cut-offs to assess the perpetuating factors for individualized treatment* | | | |
| --- | --- | --- | --- |
| **Treatment module** | **Instrument** | **Outcome** | **Cut-Off^a^** |
| *Coping with cancer and treatment* | Impact of Event Scale^b,c^ | Intrusive thoughts | ≥10 |
|  |  | Avoidance | ≥10 |
| *Fear of cancer*  *recurrence* | Modified Cancer Acceptance Scale^c,g^ | Fear of disease recurrence | ≥7 |
|  | Cancer Worry Scale^c,g^ | Worries about the risk of developing cancer again | ≥14 |
| *Helpful thinking* | Modified Causal Attribution List^c,g^ | Attribution to cancer-related factors | N/A |
|  |  | Attribution to physical factors | N/A |
|  |  | Attribution to psychological factors | N/A |
|  |  | Attribution to other sources of distress | N/A |
|  | Illness Management Questionnaire^c^ | Focusing on symptoms | ≥30 |
|  | Fatigue catastrophizing scale^c^ | Catastrophizing in response to CRF | >16 |
|  | Self-Efficacy Scale^c^ | Self-efficacy regarding CRF | ≤19 |
| *Sleep-wake rhythm* | Sleep-Wake-Diary^g^ | Protocol of individual sleep-wake-rhythm | N/A |
|  | Sickness of Impact Profile, subscale sleep & rest^d^ | General report on sleep and rest patterns | >60 |
|  | Groningen Sleep Quality Scale^f,g^ | Sleep quality | N/A |
| *Activity regulation* | Activity pattern (interview) | Relatively active vs. low active | N/A |
|  | Sickness of Impact Profile, subscale social activity^c^ | Social activities | >150 |
|  | Checklist Individual Strength, subscale concentration problems^e^ | Mental activities | > 18 |
| *Social support* | Van Sonderen Social Support Inventory^c^ | Discrepancy between actual and desired support (SSLD) | ≥50 |
|  |  | Negative Interactions (SSLN) | > 10 |
| ^a^cut-offs were taken from previous projects conducted with this program; ^b^ in this instrument, the term event was replaced by ‘cancer disease and related treatment’; ^c^ for source see Abrahams et al. (DOI: 10.1186/s12885-015-1787-7); ^d^ Hütter et al. (DOI: 10.1080/08870449708407396); ^e^ Worm-Smeitink et al. (DOI: 10.1016/j.jpsychores.2017.05.007); ^f^ Jafarian et al. (DOI:10.1016/j.sleep.2007.06.017); ^g^ questionnaire was translated from Dutch into German | | | |

| **Table S2.** *Additional deviations from study protocol as described at ClinicalTrials.gov (number: NCT03968250)* | | |
| --- | --- | --- |
| **Domain** | **Topic** | **Details and rationale for deviation** |
| **Eligibility criteria** | *Fatigue* | We initially planned to use the EORTC-QLQ C30 fatigue subscale questionnaire to select patients with high CRF, but then used the CIS-Fat to ensure maximum comparability with previous studies on this program (e.g., Abrahams et al., 2017) |
| **Design** | *Sample size* | We initially planned to recruit 20 patients, but could only recruit 17 patients. |
|  | *Screening procedure* | At study start, all interested patients were directly screened in person for both psychological and medical eligibility criteria. During the recruitment phase, we developed an online pre-screening in order to reduce patient burden. This pre-screening assessed the psychological core criteria depression and CRF. Only patients that passed this pre-screening underwent the complete in person screening. |
| **Outcomes** | *Depressive symptoms* | Given that the previous RCT of this online-program among breast cancer assessed psychological distress (Abrahams et al., 2017), we added depressive symptomatology to improve comparability. |
|  | *Patient satisfaction* | In addition to the WAI-SR, we also assessed the ZUF-8. |
